# Invasion timing affects multiple scales, metrics, and facets of biodiversity outcomes in ecological restoration experiments

**DOI:** 10.1002/eap.70062

**Published:** 2025-06-18

**Authors:** Emma Ladouceur, Michael Wohlwend, Michele R. Schutzenhofer, Jonathan M. Chase, Tiffany M. Knight

**Affiliations:** ^1^ Department of Biology University of Prince Edward Island Charlottetown Prince Edward Island Canada; ^2^ Canadian Centre for Climate Change and Adaptation University of Prince Edward Island St. Peter's Bay Prince Edward Island Canada; ^3^ School of Climate Change and Adaptation University of Prince Edward Island Charlottetown Prince Edward Island Canada; ^4^ German Centre for Integrative Biodiversity (iDiv) Halle‐Jena‐Leipzig Leipzig Germany; ^5^ Wildlife Ecology and Management Albert‐Ludwigs‐Universität Freiburg Freiburg im Breisgau Germany; ^6^ Division of Science and Mathematics McKendree University Lebanon Illinois USA; ^7^ Department of Computer Science Martin Luther University Halle‐Wittenberg Halle (Saale) Germany; ^8^ Department of Community Ecology Helmholtz Centre for Environmental Research – UFZ Halle (Saale) Germany; ^9^ Institute of Biology Martin Luther University Halle‐Wittenberg Halle (Saale) Germany; ^10^ National Tropical Botanical Garden (NTBG) Kalaheo Hawaii USA

**Keywords:** biodiversity change, disturbance, diversity, grassland restoration, Hill numbers, rarefaction, scale dependence

## Abstract

The need to develop optimal restoration protocols for degraded grasslands has led to experimental research aimed at determining how different restoration treatments influence outcomes for biodiversity. The magnitude and direction of diversity responses to restoration treatments may depend on the spatial scale at which diversity is measured (local, regional), the metric of diversity used (Hill numbers), and the facet of diversity (taxonomic, functional, phylogenetic) considered. We conducted a long‐term factorial experiment in a degraded grassland in Missouri, USA, in which we experimentally applied a regionally appropriate biodiverse seed mixture, added soil nutrients to restore soil fertility, and varied the timing in which the invasive plant *Lespedeza cuneata* entered the community. We used a unified framework of Hill numbers to evaluate how treatments influenced diversity, considering different spatial scales, metrics, and facets (taxonomic, phylogenetic, functional). We find that the timing in which the invasive *L. cuneata* entered the community had large effects on diversity, while nutrient addition had more limited effects. This was driven by the high dominance of the focal invasive when allowed to invade early in the growing season, suppressing diversity. The positive effects of late invasion increased in magnitude with spatial grain and were higher for taxonomic than phylogenetic and functional facets of diversity. This was largely due to the dominance of the focal invasive, negatively affecting diversity within specific plant families or functional phenotypes across treatments. Under early invasion, nutrients had a negative effect, particularly at local scales, inflating beta diversity in this treatment and resulting in negative to no effect of late invasion on many aspects of beta diversity. Our results demonstrate the importance of looking at a multitude of different measures of diversity to understand the relative effects of ecological restoration treatments combined with invasion timing. Efforts to keep noxious plant invaders out of a system early in restoration approaches better allow desirable, native plants to establish and can have long‐term benefits for multiple aspects of diversity.

## INTRODUCTION

As the human impact on and degradation of biodiversity continues to expand, it becomes increasingly crucial for ecologists to comprehensively quantify and understand the full extent of the effects of human‐related disturbances such as nutrient depletion or pollution, invasive species, and land degradation so that effective action can be taken (Díaz & Malhi, 2022; Magurran & McGill, 2010). Quantifying diversity across different scales, metrics, and facets allows us to better identify the most damaging drivers of diversity change (Gonzalez et al., 2023), develop predictions about future changes, and devise actions for conserving and restoring diversity (Leclère et al., 2020; Loreau et al., 2022). The field of restoration ecology aims to create conditions that allow degraded landscapes to transition from degraded conditions to states with higher levels of diversity and ecosystem functioning (Carlucci et al., 2020; Ladouceur et al., 2022; Temperton et al., 2019) and has become increasingly relevant as international agreements have declared restoring degraded habitats as a priority (UNEA, 2019).

While simply ceasing the activities that cause habitat degradation is an important part of habitat restoration, this is often not enough (Ladouceur et al., 2023; Temperton et al., 2004). First, the abiotic conditions of degraded habitats (e.g., soil chemistry, nutrient availability) often require amelioration to allow more natural communities and ecosystems to recover and develop (e.g., Bischoff et al., 2009; Sáez‐Sandino et al., 2023). Likewise, exotic invasive species often become dominant in degraded habitats, and removing the drivers of degradation alone is often not enough to allow the native community to rebound without simultaneous direct management of the established exotic invasive species (Crandall & Knight, 2015; Weidlich et al., 2021; Young et al., 2017; Yu et al., 2020). Thus, the complexity of developing optimal restoration protocols has led to experimental work aimed at understanding how manipulations of abiotic conditions and exotic invasive species management can best restore diversity (Brudvig, 2011; Humphries et al., 2022; Wohlwend et al., 2019).

Rather than being a single quantity, biodiversity and diversity are catchall terms that describe dozens of metrics that quantify different aspects of numbers and types of species. Biodiversity metrics do not always respond to ecological drivers by the same magnitude or even direction at different spatial and temporal scales (Blowes, Daskalova, et al., 2022; Chase et al., 2018, 2019). Therefore, it is important to consider a variety of diversity features when evaluating diversity change in response to global change drivers. This includes (1) spatial scale: species diversity increases non‐linearly with increasing sampling effort (or area), and biodiversity change depends on the scale at which diversity is measured/observed, the species abundances, and spatial distributions at that scale (Chase et al., 2018; He & Legendre, 2002; McGill, 2011; McGlinn et al., 2021). (2) Metrics: measures that explicitly incorporate species' relative abundances and give different weights to commonness and rarity (e.g., Shannon, Simpson, Hill number order *q* = 1 or 2) help to understand effective numbers of dominant/highly abundant species (Chao et al., 2021). Lastly, (3) the facets: taxonomic makeup of the assemblage (i.e., the species names), as well as other biological features, including functional traits (i.e., functional diversity; Cadotte et al., 2011), and phylogenetic relationships (i.e., phylogenetic diversity; Barber et al., 2017; Khalil et al., 2017) help to understand structural relationships from different perspectives. Leveraging these aspects, we can better quantify and understand the multifaceted nature of the effects of disturbance and restoration on diversity.

Drivers of biodiversity change can affect different scales, metrics, and facets of diversity in surprisingly diverging ways. The effect of a driver can increase with scale if it reduces site‐to‐site variation in composition, causing the loss of low‐occupancy species and/or decreasing spatial clumping. Alternatively, the effect of a driver can decrease with scale if it increases local variation (Chase et al., 2018, 2019). For example, in a ~30‐year grazing experiment in Kansas tallgrass prairie, bison had a more positive effect at local scales, increasing variation and reducing dominant grasses (Ratajczak et al., 2022) and influencing local dynamics like coexistence and turnover of species. Likewise, a driver can have larger effects on patterns of species richness than metrics that weight common species more heavily if it influences rarer species, whereas similar effects emerge if the driver equally influences all species (Chao, Chiu, et al., 2014). For example, in oak‐prairie savannas in Minnesota, species richness recovered better than measures that account for community evenness after 80–100 years since agricultural abandonment due to rare species (Isbell et al., 2019; Ladouceur et al., 2023). Finally, if a driver has a larger effect on taxonomic diversity than on functional or phylogenetic relationships, species responses might have been mediated by their traits and relationships. For example, in restored grasslands in Illinois, the time since restoration negatively affected plant taxonomic diversity but positively influenced functional and phylogenetic diversity at local scales (Guiden et al., 2021). In restoration, small scales are relevant for understanding regeneration and local community dynamics, and larger scales are relevant for understanding the big picture, the ultimate goal of restoration outcomes worldwide. Abundance weightings are valuable for understanding dominance and rarity in reassembly, and facets reveal if treatments affect particular families or functional phenotypes differentially.

In many parts of the world, as in North American prairies, native grasslands have been degraded by agricultural activities that deplete soil nutrients and then are often invaded by exotic plant species (Humphries et al., 2021; Kindscher & Tieszen, 1998). When establishing restoration protocols for these habitats, abiotic conditions (e.g., soil nutrients) and local exotic invasive species must be considered concomitantly (Bardgett et al., 2021). In this study, we focused on different actions relevant to restoration on a nutrient‐poor prairie recovering after agriculture, prone to invasion by a common exotic N‐fixing legume, *Lespedeza cuneata*. We implemented a factorial restoration experiment that seeded a diversity of native grasses and forbs, manipulated soil nutrients, as well as the timing of native species seeding and *L. cuneata* invasion (Wohlwend et al., 2019). *L. cuneata* is an N‐fixer and a fast‐growing but long‐lived perennial that rapidly spreads both by rhizomes and seeds. We hypothesized that the invaders dominance would be reduced when nutrients were added to the system and even further if native species were able to establish first (Weidlich et al., 2021). We confirmed in a previous study that the late invasion timing treatment suppressed the abundance of *L. cuneata* in a way that persisted for 7 years and resulted in different compositions of species across treatments (Wohlwend et al., 2019). We now compare these treatments to focus on the relative success of each for diversity to inform applied management actions relevant to ecological restoration.

Here, we consider the relative responses of multiple facets of diversity to these factorial treatment combinations aimed at restoring native diversity and suppressing a problem invader in the same 7‐year‐long experiment. Specifically, we asked the following questions: (1) Is there spatial‐scale dependence in the diversity response to adding nutrients and/or the timing of invasion by *L. cuneata*? We addressed this by comparing the responses of multiple diversity components measured at the small, within‐plot scale, as well as the accumulation of diversity across all plots within a treatment. (2) Do diversity metrics vary in their responses to the addition of nutrients and/or the timing of invasion? We addressed this by calculating metrics that differentially weight rare and common species (i.e., Hill numbers, Hill, 1973, as emphasized by Chao, Gotelli, et al., 2014; Jost, 2006). (3) Do diversity facets vary in their responses to the addition of nutrients and/or the timing of invasion? We addressed this by quantifying metrics that include information on the taxonomic, functional, and phylogenetic diversity. By knowing the interactive effects of management actions across scales, metrics, and facets, we can better understand the outcomes relevant to ecological restoration.

## METHODS

### Study system

Our experiment was conducted in a 0.5‐ha field located at the Tyson Research Centre of Washington University in Missouri, USA (38°30′31.1″ N and 90°34′21.6″ W). The area has a warm and temperate climate with an annual precipitation of 897 mm and an average annual temperature of 13.7°C. The soils are derived from limestone and are rich in clay. The study area was used as an experimental corn field from 1984 to 1989, after which it was left fallow with intermittent mowing to prevent tree invasion. Prior to our experiment, *L. cuneata* was found in the site and in areas around it (more details in Wohlwend et al., 2019).

To eliminate *L. cuneata* from the site and prepare the area for our experimental restoration treatments, we applied broadcast treatments of 40% glyphosate herbicide (July 2007, June 2008). Additionally, the field was mowed in July 2008, disked in August 2008, and tilled in February 2009 to deplete the seed bank of *L. cuneata*. The field was managed with common prairie restoration practices used in the region, detailed below, throughout the experiment, which took place from 2009 to 2016. In June and August 2009, the entire field was mowed to remove dead biomass. In 2011, 2013, and 2016, late winter or early spring burns were conducted, which are part of the natural disturbance regime of prairies. Three non‐native species that are known to exhibit invasive behavior, *Carduus nutans*, *Vicia villosa*, and *Sorghum halepense*, were either manually removed or spot‐targeted with a 40% glyphosate herbicide.

### Focal invasive species

*L. cuneata*, commonly also known as *Sericea lespedeza*, is a perennial plant in the family Fabaceae. It is native to China, Japan, and Korea but has been widely introduced for erosion control and as a forage crop, which is able to grow in nutrient‐poor soils and harsh conditions (Ohlenbusch et al., 2007). It has become naturalized in many parts of the world, including the United States. Young plants provide valuable forage, but mature plants have a high concentration of condensed tannins and lignin and are avoided by grazers (Tracy et al., 2022). The plant grows to be approximately a meter tall, with small white or pink flowers that bloom from July to September. Plants produce new sprouts arising from root nodes, and many small seeds can last in the soil seed bank for several years (Ohlenbusch et al., 2007). Numerous factors are thought to contribute to the invasiveness of *L. cuneata*, such as its ability to thrive in nutrient‐poor soils, its rapid growth, its allelopathy, and enemy release (Allred et al., 2010; Kalburtji et al., 2001; Schutzenhofer et al., 2009; Schutzenhofer & Knight, 2007).

## EXPERIMENTAL DESIGN

### Treatments

In 2009, we established one hundred and two 10‐m^2^ (3.16 m × 3.16 m) restoration plots in a 7 × 15 cell design, with a 2‐meter separation between plots. The field was split into four blocks that represented a slight moisture gradient. We studied the effects of seeding native species, nutrient addition, and invasion timing on forb diversity to overcome nutrient depletion and invasion typical of degraded prairies in the region. Each plot received a random combination of three treatment categories: nutrients, manipulated invasion timing, and assembly of seeded forbs and grasses. In 2009, all plots were hand‐broadcasted with 25 Missouri ecotype native forb and 5 grass species typical of prairie restorations in the region. We initially considered an additional treatment of “assembly” to vary the order of forb and grass seeding, and while this treatment had effects on the invader (Wohlwend et al., 2019), it had minor effects on forb diversity and thus was excluded from presentation here for clarity (see *Data preparation* for more detail). The nutrient treatments were control (not fertilized) and nutrients added, with the latter plots receiving a slow‐release fertilizer (Scotts Osmocote Classic) containing 6 gNm^−2^ year^−1^, 2.7 g ammoniacal nitrogen, 3.2 g nitrate nitrogen, 2 g phosphorus (as P_2_O_4_), and 4 g potassium (as K_2_O), applied annually in June from 2009 to 2015. The invader was seeded by hand into experimental plots, once in 2009 (early treatment), at the same time as seeding native forbs and grasses, and once in 2012 (late invasion treatment). The late invasion treatments required the use of spot spraying of emerging *L. cuneata* with glyphosate to maintain its integrity until seeding 2012. All plots were invaded successfully.

### Plant community surveys

In 2016, we comprehensively sampled the plant community within each plot. To achieve this, we established a grid of nine subplots within each plot, each subplot measuring 0.5 by 0.5 m (0.25 m^2^), with a buffer of 0.5 m between each subplot. This resulted in 864 subplots across 102 plots, equaling 72 subplots per treatment combination. Within each of these subplots, we carefully documented the identity of each forb species and identified grasses as a single group to estimate the percentage of ground cover that each represented visually. As a result, here we focus on forb diversity as a measure of interest for prairie restoration in this region. After the sampling of all nine subplots was complete, we continued the survey by walking the perimeter of the plot. Our objective was to identify any rare forb species that were present within the plot but not observed in the subplots. These rare species were documented and recorded as present to ensure a complete inventory of the forb plant community within each plot.

### Data preparation

We standardized plant taxonomy nomenclature using The Plant List version 1.0 in 2022 (Knight & Ladouceur, 2025; The Plant List, 2013). All data preparation and wrangling, metric quantification, statistics, and visualization of results described and presented below were conducted in the R for Statistical Computing and Graphics environment (v.4.2.3; Ladouceur, 2025; R Core Development Team, 2019). Data wrangling was conducted using the tidyverse package (Wickham et al., 2019) and visualization in ggplot2 (Wickham, 2009).

For these analyses, we considered four unique treatment combinations (2 nutrient × 2 invasion timing) and only used treatment plots where grasses and forbs were seeded together from the assembly treatment (Knight & Ladouceur, 2025). We considered the rare species perimeter walk as a tenth subplot for every plot (see more details below). This resulted in 32 plots and 320 subplots for presentation here. This amounts to 80 subplot samples per unique treatment combination. We removed the assembly treatment from the presentation here for clarity, as this treatment affected outcomes in the cover of our focal invader (Wohlwend et al., 2019) but did not affect diversity outcomes. Therefore, it brought the treatment combinations from 12 down to 4, allowing clearer presentation and understanding of main results important for forb diversity outcomes.

### Functional traits

We requested data on 11 continuous plant and regeneration traits for 122 standardized forb species names from the TRY global plant trait database (Kattge et al., 2011). We requested trait data for commonly studied traits found to be important for the leaf economic (Wright et al., 2004), the global plant (Díaz et al., 2016; Reich, 2014), and the regeneration trait spectrum (Fernández‐Pascual et al., 2021; Ladouceur et al., 2019). Regeneration traits are important to the trajectory and functioning of restored systems (Cadotte et al., 2011). Overall, we obtained species‐level values for the following traits: leaf area (in square millimeters, LA), specific leaf area (in square millimeters per milligram, SLA), leaf dry matter content (in grams of dry mass per gram of fresh mass, LDMC), leaf nitrogen (in milligrams per gram, LN), vegetation plant height (in meters, PH), soil seed bank longevity (in years, SSBL), seed dry mass (in milligrams, SDM), seed germination rate (in percentage, SGR), seed number per reproduction unit (number, SN), stem specific density (in grams per cubic centimeter, SSD), and plant vegetative reproduction: lateral spread (PVR). Plant vegetative reproduction did not have enough data coverage for the species list we submitted, so we did not include it in our analyses and used 10 traits for our final trait dataset. We used standardized units for single traits and best estimate measurements from the TRY database for all species and traits requested and took the mean trait value for each species and each trait where multiple values were returned.

### Phylogenetic trees and trait imputation

We generated a phylogenetic tree for our list of species based on a backbone plant mega phylogenetic tree based on the botanical nomenclature of the World Plants (WP database; https://worldplants.de), using V.PhyloMaker, which generates multiple trees based on various established methods (Jin & Qian, 2019; Smith & Brown, 2018; Zanne et al., 2014). We used the third default phylogenetic scenario generated, in which the tip for a new genus is bound to the 1/2 point of the family branch unless the family branch is longer than 2/3 of the whole family branch length and the tip of the new genus is bound to the upper 1/3 point of the whole family branch length (Jin & Qian, 2022).

We estimated the proximity of tips of our phylogeny using Abouheif's proximity without diagonal and no normalization. Based on this phylogenetic distance matrix, we quantified Moran eigenvectors and used the first 30 values; we then used this phylogenetic correlation structure, our list of species, and their traits to predict missing trait values (Debastiani et al., 2021; Penone et al., 2014). We predicted missing trait values by performing nonparametric missing value imputation for species and their traits using random forest models (Penone et al., 2014; Stekhoven & Buhlmann, 2012). The eigenvectors contribute to this by eliminating features that have a strong correlation between them to help reduce the overfitting of imputed values (Penone et al., 2014). We re‐iterated the estimation of phylogenetic eigenvectors 25 times for 100 random forest trees with variable‐wise imputation. We selected values for traits and species that minimized out‐of‐bag (OOB) imputation error, a measure of prediction error in random forest models, for each species and trait. We took the number of eigenvectors that minimized these errors for the species traits of interest. Phylogenetically corrected trait imputation was quantified using the adephylo (Jombart et al., 2010; Jombart & Dray, 2008) and the missForest packages (Stekhoven, 2022; Stekhoven & Buhlmann, 2012).

### Quantifying diversity

We quantified forb diversity for two metrics (Hill numbers *q* = 0 and *q* = 2) across multiple scales and for three facets of diversity (taxonomic, phylogenetic, functional) (Chao et al., 2021; Chao, Chiu, et al., 2014; Chao, Gotelli, et al., 2014; Hill, 1973). When conducting diversity surveys in grasslands, recording species incidence (detection/non‐detection) is more pragmatic than quantifying the number of individuals (abundance) (Chao et al., 2021), so here we have used sample‐based rarefaction with incidence data to quantify forb diversity appropriate for our mesic grassland system.

Novel approaches with hill numbers provide unified measures of diversity within assemblages, where the order *q* determines the sensitivity of the measure to the (incidence‐based) relative abundance of species (Chao et al., 2021; Hill, 1973). To assess diversity across restoration treatments, we focus on Hill numbers *q* = 0 and 2 to unify three indices of diversity. Taxonomic diversity (TD) of *q* = 0 reduces to species richness, and TD of *q* = 2 reduces to Simpson diversity, which can be interpreted as the effective number of dominant or highly abundant species.

To unify Hill numbers with phylogenetic (PD) and functional (FD) facets of diversity, we used an attribute‐diversity approach (Chao et al., 2010, 2019, 2021). For example, a taxonomic attribute represents a species in TD, whereas a phylogenetic attribute represents an effective number of equally divergent lineages (meanPD) in our selected phylogenetic tree (default third scenario) for PD (Chao et al., 2010). An effective number of equally divergent lineages is quantified as the effective total branch length divided by the tree depth (Hu & Chao, 2022). A functional attribute represents a virtual functional group in FD (Chao et al., 2021), quantified as the FD under specified threshold values (tau values). We set our tau values threshold to NULL, which is quantified as the mean distance between any two individuals randomly selected from the pooled assemblage (Hu & Chao, 2022). We quantified functional distances in our FD approach for all species and traits from our imputed trait matrix, using a species pairwise distance matrix, with Gower's dissimilarity as our distance metric (Pavoine et al., 2009).

For rarefaction of diversity estimates, we treated each subplot (0.25 m^2^, 0.5 × 0.5 m) as a sampling unit, and we quantified the “rare species survey around the perimeter of each plot” as a 10th subplot sample for each plot. We extracted the species incidence in each subplot to obtain an occurrence count (incidence‐based frequency) of each species in 80 samples for each treatment. We extrapolate estimates out to 120 subplot samples and quantify 95% CIs based on 50 bootstrap samples for every sample size across rarified (<80), observed (80 samples), and extrapolated (>80) numbers of sampling units. Each treatment combination selected for this analysis contained at least 80 subplots nested within 32 plots.

For all quantification of Hill numbers, sample‐based incidence occurrence rarefaction, and extrapolation for all facets of diversity, we used the “iNEXT.3D” package (Hu & Chao, 2022). To quantify Gower's dissimilarity and produce a distance matrix based on our imputed traits for the estimation of FD, we used the cluster package (Maechler et al., 2022).

### Assessing differences in diversity

To quantify the effect of restoration treatments on diversity, we focus on two spatial scales. Our smallest alpha‐scale (hereafter α‐scale) is two subplots (0.5 m^2^) because, for incidence‐based rarefaction where order *q* = 2, we required a minimum of two samples to account for abundance. Second, our large (hereafter γ‐scale) is 80 subplots (20 m^2^), the total number of observed subplots within treatments. To quantify the relative effect of Lespedeza invasion timing on each scale (α, γ), each metric (*q* = 0, *q* = 2), and each facet (TD, PD, and FD), we took rarefied diversity estimates at our α‐scale, within two subplots, and observed diversity estimates at our γ‐scale for every metric and facet. From α and γ estimates, we estimated Whittaker's multiplicative β‐diversity (Whittaker, 1972) (β = γ/α) to quantify subplot variation or the heterogeneity of subplots within treatments. We then subtracted estimates in “early invasion” treatments from estimates in “late invasion” treatments for the mean and CIs to quantify an effect size and confidence around each treatment effect. Lastly, we plot diversity estimates of all four treatments and their CIs against one another for comparison of treatment effects on each scale, metric, and facet.

## RESULTS

**FIGURE 1:**
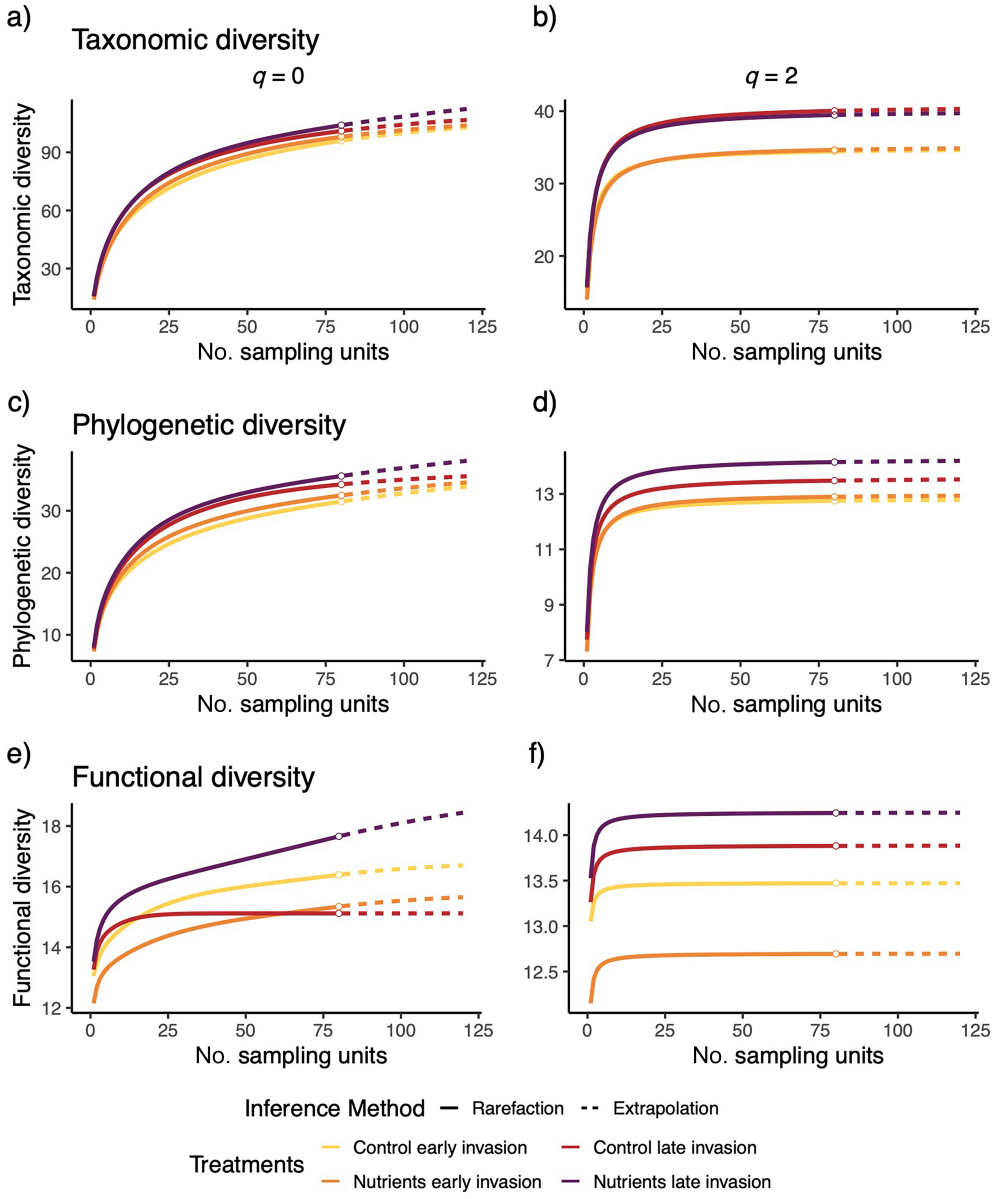
Sample‐based rarefaction (solid line) and extrapolation (dotted line) for *q* = 0 and *q* = 2–120 subplots (0.25 m^2^ each) for forb diversity. Top row (a, b) is taxonomic diversity, middle row (c, d) is phylogenetic diversity, and bottom row (e, f) is functional diversity. Left column is *q* = 0, and right column is *q* = 2. Colors denote four different factorial treatment combinations. CIs are not shown for clarity but can be found in Appendix S1: Table S1 and are visually represented in Figures 2, 3, 4. *Y*‐axes are varied for clarity.

**FIGURE 2:**
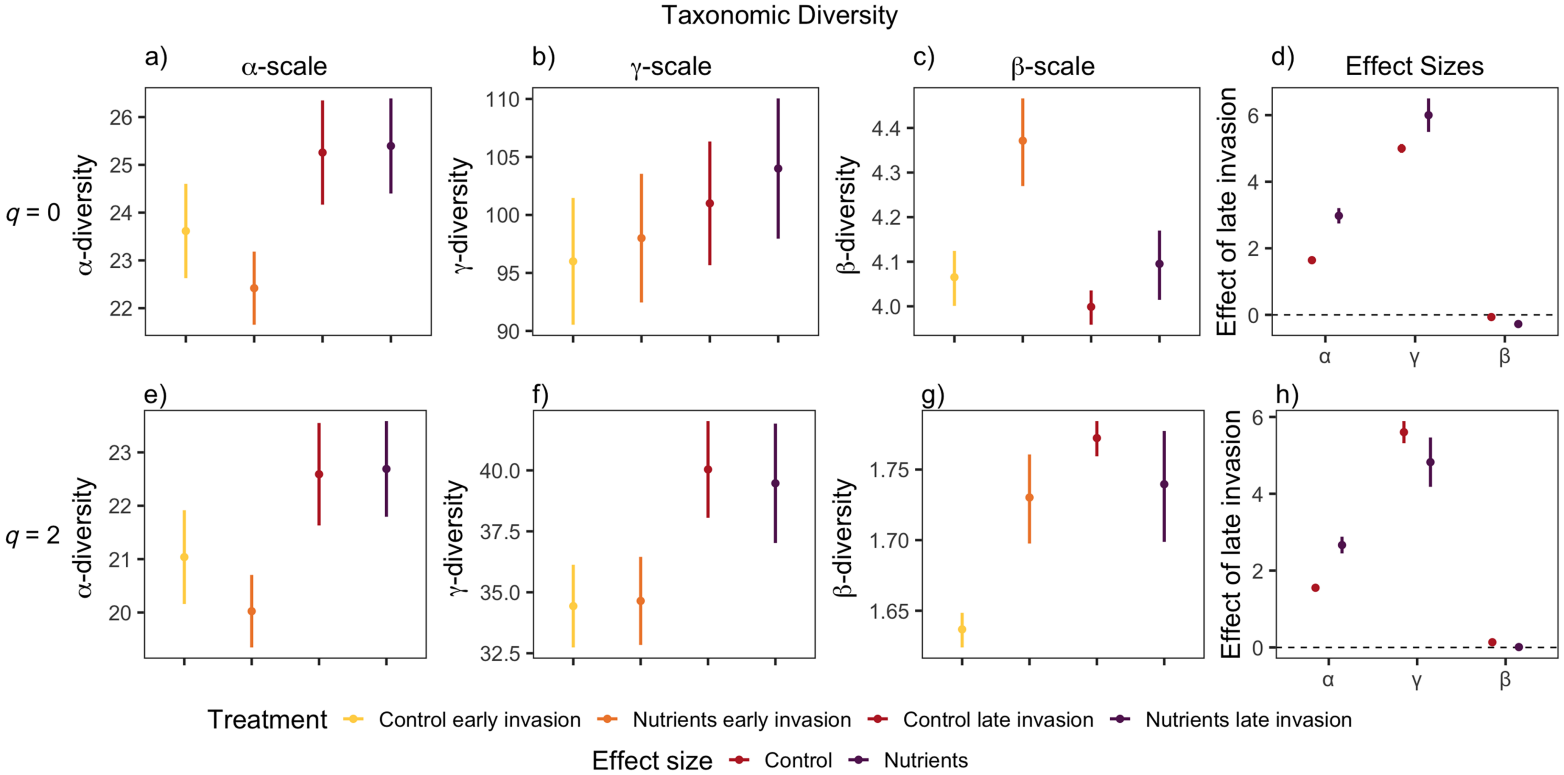
Taxonomic diversity values associated with Figure 1. In (a) and (e): α‐scale (2 subplots), in (b) and (f): γ‐scale (80 subplots), and in (c) and (g): Whittaker's β‐scale (γ/α) diversity for *q* = 0 (first row) and *q* = 2 (second row). In (d) and (h): the effect of invasion timing (late–early) of *Lespedeza cuneata* on forb diversity within nutrient treatments associated with the row it is in line with. Points indicate mean effect; lines indicate 95% CIs. Colors denote factorial nutrient treatments. *Y*‐axes are varied for clarity.

**FIGURE 3:**
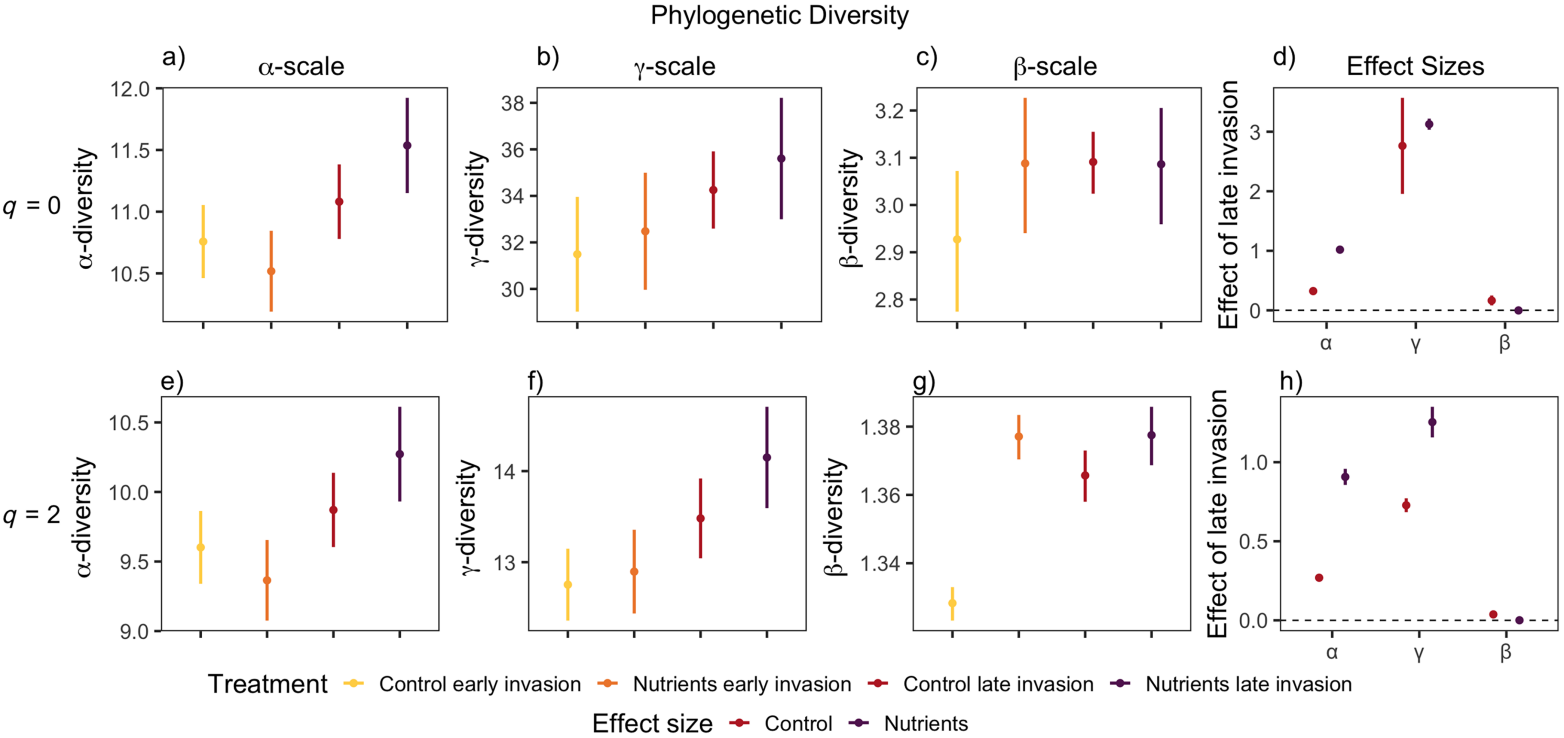
Phylogenetic diversity values associated with Figure 1. In (a) and (e): α‐scale (2 samples), in (b) and (f): γ‐scale (80 samples), and in (c) and (g): Whittaker's β‐scale (γ/α) diversity for *q* = 0 (first row) and *q* = 2 (second row). In (d) and (h): the effect of invasion timing (late–early) of *Lespedeza cuneata* on forb diversity within nutrient treatments associated with the row it is in line with. Points indicate mean effect; lines indicate 95% CIs. Colors denote factorial nutrient treatments. *Y*‐axes are varied for clarity.

**FIGURE 4:**
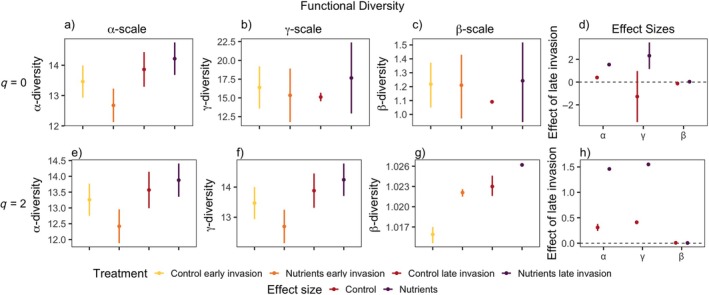
Functional diversity values associated with Figure 1. In (a) and (e): α‐scale (2 samples), in (b) and (f): γ‐scale (80 samples), and in (c) and (g): Whittaker's β‐scale (γ/α) diversity for *q* = 0 (first row) and *q* = 2 (second row). In (d) and (h): the effect of invasion timing (late–early) of *Lespedeza cuneata* on forb diversity within nutrient treatments associated with the row it is in line with. Points indicate mean effect; lines indicate 95% CIs. Colors denote factorial nutrient treatments. *Y*‐axes are varied for clarity.

Overall, we found that at both α‐ and γ‐scales, for all facets of diversity, the late invasion of the invasive species *L. cuneata* had the most positive effect on diversity compared to other treatments, especially when combined with nutrient addition (Figure 1a–f, Appendix S1: Table S1). Additionally, across both metrics (*q* = 0, *q* = 2) and all facets (TD, FD, and PD) of diversity, we found that the effect size of the timing in which *L. cuneata* invaded the community generally increased with the increasing spatial scale in which diversity is measured, except for functional diversity when common and rare species were weighted equally (Figures 2, 3, 4). The effect of late invasion for control and nutrients was positive overall for small and large scales, across all facets and metrics of diversity, but notable exceptions emerged. The positive effect of late invasion was largest on taxonomic diversity (Figure 2). Rare species drove the positive effect of nutrients on late invasion at larger scales (γ‐scale), as the effect disappeared when metrics were corrected for more abundant species (*q* = 2) (Figure 2). While functional diversity was the least affected by our treatments, the combination of late invasion and nutrient addition had a positive effect on phylogenetic and functional diversity at the small and larger scales for both *q* = 0 and *q* = 2 (Figures 3 and 4). This indicates a positive result for rare and more abundant species identities, phylogenetic relationships, and functional structure overall.

For taxonomic diversity (Figure 2), the effect of late invasion increased diversity at order *q* = 0 (equivalent to species richness) at small and large scales, more so at large scales (a, b, d). However, Whittaker's β‐diversity (*q* = 0) was negatively affected by late invasion and nutrient addition because α‐diversity was lowest under nutrient addition (a) and early invasion, leading to the largest ratio between the large and small scales (c, d). Late invasion and nutrients affected the effective number of dominant species positively at the α‐scale (a, h) but negatively at the γ‐scale compared to the control (f, h). This positive difference due to nutrients is only due to diversity being so low under early invasion and nutrient addition (a, e), also inflating β‐diversity for this treatment (c, g). Late invasion positively increased more evenly abundant species (order *q* = 2) (h). β‐diversity saw a positive increase under late invasion in the control treatment but no effect under nutrient addition (g, h), as β‐diversity was similarly high in both treatments (g).

For phylogenetic diversity (Figure 3), the effect of late invasion increased diversity at order *q* = 0 at small and large scales (a, c, d) for rare and common species (e, f, h). Nutrient addition added to this effect at small scales for *q* = 0 (a, d) and at small and large scales for abundant species (e, f, h). Phylogenetic β‐diversity responded positively to late invasion in the control but had no effect under nutrient addition for both rare and common species (d, h), as β‐diversity was also high under early invasion with nutrients due to very low α‐diversity in this treatment (c, g).

Functional diversity (Figure 4) was affected similarly positively by late invasion at small scales (a, e) for both orders *q* = 0 and 2 (d, h). At larger scales, nutrient addition affected *q* = 0 but negatively in the control treatment (b, d), but for *q* = 2, more equally abundant functional phenotypes were affected positively at larger scales by late invasion and notably by nutrient addition (f, h). Functional β‐diversity saw no effect of late invasion under nutrient addition and had a negative effect in the control treatment for *q* = 0 (c, g, d, h). Rare species largely drove this negative effect, as β‐diversity was affected very little by late invasion for *q* = 2 in controls lacking nutrient addition (h). Full results are found in Appendix S1: Tables S1–S3.

## DISCUSSION

It has become more evident in recent years that different ways of looking at diversity are important to be considered to gain a more complete picture of the multifaceted nature of diversity change. In this study, we found that comparative responses of diversity to restoration treatments are strongly influenced by the spatial scale in which diversity is measured, the weighting of diversity measures to common versus rare species, and which facet of diversity (e.g., functional and phylogenetic) is measured. We found that treatments manipulating invasion timing and nutrient addition in former agricultural fields, vulnerable to an invader, are relevant not only for invader cover and community composition (Wohlwend et al., 2019) but also to the multifaceted aspects that encapsulate diversity. We found that the most dramatic results are observed for the effect of invasion timing at larger spatial scales, particularly for taxonomic diversity.

Across both metrics (*q* = 0, *q* = 2) and all facets (TD, FD, and PD) of diversity, we found that the effect of late *L. cuneata* invasion generally increased with spatial scale, except in the case of functional diversity when common and rare functional phenotypes were weighted equally (see below). The majority of our understanding of the effects of different drivers on grassland communities comes from small‐scale plots similar in size to those that we used here at our α‐scale (e.g., 1 m^2^) (Isbell et al., 2019; Ladouceur et al., 2023). However, it is clear that the outcomes depend critically on the spatial scale in which grassland diversity is measured, and effects can either be magnified or reversed at larger scales (Chalcraft et al., 2008; Ladouceur et al., 2023; Seabloom et al., 2021). Larger scales can add valuable perspectives to restoration approaches, given that the goals of restoration are aimed at large‐scale beneficial effects and the big picture.

When effect sizes increase with increasing scale, it implies that the community is becoming more homogenous in the presence of a given driver (in this case, timing and subsequent dominance *L. cuneata*), and β‐diversity is lower (Chase et al., 2018, 2019; Chase & Knight, 2013). Homogenization is a frequently suggested response to the presence of dominant invasive plants (Stotz et al., 2019). This can occur if species that are relatively low in occupancy (e.g., rare species) decrease in their occupancy or if species that are relatively higher in their occupancy (e.g., widespread and invasive species) increase their abundance (Blowes, McGill, et al., 2022; Socolar et al., 2016). In this case, *L. cuneata* became dominant in the early invasion treatment, especially when nutrients were added (Wohlwend et al., 2019), reducing α‐scale diversity. Native species increased in the late invasion treatment, showing more plot‐to‐plot turnover in their diversity, leading to a negative to little effect of late invasion on β‐diversity relative to comparative treatments. The dominance of the invader contributed to the suppression of particular plant families and functional phenotypes, lowering phylogenetic and functional diversity compared to taxonomic measures. For example, some important seeded species that are indicative of native prairies, such as *Echinacea* spp., were able to establish in the late, but not early, invasion treatment.

While we observed homogenization in the face of invasive *L. cuneata* dominance and higher effect sizes of invasion timing on the γ‐scale compared to the α‐scale, this is by no means the only outcome that can occur in the face of intense invasions. For example, Powell et al. (2011, 2013) showed that the effects of invasive species on diversity can be intense within local plots when the invasive dominates but much less intense at the γ‐scale, as often individuals of many native species can be present across replicate plots. Likewise, it has been suggested that in many cases, invasive species can instead create differentiation among local communities when low‐occupancy species increase and/or high‐occupancy species decrease in abundance (Blowes, McGill, et al., 2022; Socolar et al., 2016). In this case, effect sizes can even decrease with increasing scale.

Importantly, when we compared effects on each of the diversity metrics when common and rare species were weighted equally (*q* = 0), we found similar effects to when common and dominant species were given much greater weight (*q* = 2) along the Hill number continuum in most cases. This suggests that the effects of invasion timing had equal effects on both the most common species in the community, like those discussed above, as well as several of the rarer species in the community that only make up a small percentage of the total community (but a much larger percentage of species richness estimates). In cases where these metrics differed, it was due to a combination of differences in rare and common species under early and late invasion, usually due to diversity being affected more negatively under early invasion and nutrient addition.

We found that taxonomic diversity showed a much greater response to the invasive timing treatment than both phylogenetic and functional diversity. For phylogenetic diversity, for example, species in the mint family (Lamiaceae) were 10 times more abundant in the late invasion treatment than the early invasion treatment (Appendix S1: Table S4). On the other hand, species in the legume family (Fabaceae) were more common in the early invasion treatment, almost entirely due to the dominance of our focal invader, *L. cuneata* (Appendix S1: Table S4). Despite these shifts, phylogenetic diversity mostly remained similar between the invasion timing treatments. For example, in the early invasion treatment, the mint *Teucrium canadense*, which is indicative of disturbed habitats, was common, whereas in the late invasion treatment, *T. canadense* was present, but so were other native mints including *Monarda fistulosa*, which was seeded into the plots and an indicator species in prairie, savannas, and glade habitats (Appendix S1: Table S4). Thus, while taxonomic diversity was higher in the late invasion treatment, which had lower abundances of *L. cuneata*, phylogenetic diversity was less affected because the same plant families were represented by more weedy relatives in the early invasion treatment that was dominated by *L. cuneata*.

Functional diversity was the least affected by our treatments. This is due to the most common plant species across both invasion timing treatments all having rather intermediate values for all the functional traits considered here rather than extreme values. Further, different species with similar extreme values were present in both invasion timing treatments. For example, both *Chenopodium album* and *Barbarea vulgaris* have high values for leaf nitrogen; the former is highly abundant in the early invasion treatment, and the latter is highly abundant in the late invasion treatment. Overall, our results show that the early invasion and dominance of *L. cuneata* into a restored prairie results in the loss of species and thus losses of taxonomic diversity. However, because there is enough redundancy in the community among species at higher levels of organization, we see less dramatic losses in phylogenetic and functional diversity.

We expected that the addition of nutrients would temper the effect of invasion timing on the abundance and dominance of the invasive *L. cuneata* because its advantage as a nitrogen‐fixing legume would be reduced. While this expectation was realized and *L. cuneata* was not as dominant with nutrient additions (see results in Wohlwend et al., 2019), the effect was not substantial enough to have a strong influence on any of the patterns of diversity that we analyzed here. At the α‐scale, we see an unexpected result that the effect size of invasion timing is lower in plots where no nutrients were added even when *L. cuneata* was more abundant at small scales under this treatment (Wohlwend et al., 2019), and diversity was higher than under nutrient addition. However, this is because the effect of late invasion combined with nutrient addition was very positive at large scales, indicating that native species can still coexist with problem invaders through plot‐to‐plot variation. Even if the effects of an invader are devastating at small scales, large‐scale variation helps create refuges that could help rescue diversity under future adaptive management actions.

## IMPLICATIONS AND CONCLUSIONS

Biodiversity policy is shifting in focus from being purely about the preservation of intact habitats to recognizing that restoration of formerly degraded habitats is an equally important goal (Possingham et al., 2015). Restoration must be done right to be effective for diversity outcomes (Bekessy et al., 2010). As a result, it is important to be able to quantify just how diversity is changing in the face of degrading factors, such as the invasive species in this study, as well as in response to restoration actions that seek to enhance diversity and its contributions to ecosystem functions (Kollmann et al., 2016). Because diversity is a scale‐dependent and multifaceted concept, its quantification in the context of treatments relevant to restoration requires a scale‐explicit and multifaceted understanding as well.

We found that the spatial scale in which diversity is measured is critical for understanding how diversity responds to ecological restoration treatments that manipulated the timing in which invasive species were allowed to enter a community and nutrients. Importantly, we were much more likely to be able to detect the negative effects of the invader when diversity was quantified at much larger spatial scales than are typically measured in most grassland restoration experiments (see also Ladouceur et al., 2023). Second, while we observed a greater effect of our treatments on taxonomic diversity than on other facets, understanding impacts on other facets pointed to important compositional shifts taking place. For example, by understanding the impacts on phylogenetic diversity across scales, we better understood the impacts of a few dominating families, pointing to clear next steps in a potential adaptive management plan. In all, our results show that understanding diversity change can benefit from a multifaceted approach. Understanding how many aspects of diversity recover after agriculture, aided by treatments relevant for restoration, can help us to better apply management actions at relevant scales, targeting particular abundance dynamics across taxonomic identities, functional phenotypes, and the phylogenetic tree of life.

## AUTHOR CONTRIBUTIONS

Tiffany M. Knight and Jonathan M. Chase conceived the idea. Tiffany M. Knight, Jonathan M. Chase, and Michele R. Schutzenhofer set up the experiment. Michael Wohlwend and Michele R. Schutzenhofer conducted vegetation surveys. Tiffany M. Knight, Jonathan M. Chase, and Emma Ladouceur shaped the analyses. Emma Ladouceur conducted analyses. Tiffany M. Knight, Jonathan M. Chase, and Emma Ladouceur wrote the first draft of the manuscript, and Michele R. Schutzenhofer and Michael Wohlwend helped shape the manuscript.

## CONFLICT OF INTEREST STATEMENT

The authors declare no conflicts of interest.

## Supporting information


Appendix S1.

## Data Availability

Data (Knight & Ladouceur, 2025) are available in the Environmental Data Initiative's Data Portal at https://doi.org/10.6073/pasta/503e6ffe01cf9b94de4ac31d202f5e7a. Code (Ladouceur, 2025) to reproduce figures is available on Zenodo at https://doi.org/10.5281/zenodo.15346195.
