# Supplementary material for: Invasion timing affects multiple scales, metrics, and facets of biodiversity outcomes in ecological restoration experiments
Source: Ecol Appl. 2025 Jun 18;35(4):e70062. doi: 10.1002/eap.70062 (PMC12175749; doi:10.1002/eap.70062)
Supplement: Supplementary file 1 — Appendix S1. [file EAP-35-e70062-s001.pdf]

Emma Ladouceur, Michael Wohlwend, Michele R. Schutzenhofer, Jonathan M. Chase, Tiffany M. Knight. **Invasion timing affects multiple scales, metrics, and facets of biodiversity outcomes in ecological restoration experiments.** *Ecological Applications*

Appendix S1

Table S1: Estimates and confidence intervals for each facet of diversity, each treatment combination and at two spatial scales, a- scale (2 samples, 0.5m2) and g-scale (80 samples, 20m2). qD = Diversity at order.q, and Lower and Upper_ CI= Represents confidence intervals based on 50 bootstrap samples.

Div	Treatment	n_samples	Order.q	qD	qD_Lower_CI	qD_Upper_CI
TD	Nutrients_Early	2	q = 0	22.42	21.65	23.18
TD	Control_Early	2	q = 0	23.62	22.63	24.6
TD	Control_Late	2	q = 0	25.26	24.17	26.35
TD	Nutrients_Late	2	q = 0	25.4	24.4	26.39
TD	Nutrients_Early	2	q = 2	20.02	19.35	20.7
TD	Control_Early	2	q = 2	21.04	20.16	21.92
TD	Control_Late	2	q = 2	22.59	21.63	23.55
TD	Nutrients_Late	2	q = 2	22.69	21.79	23.59
TD	Control_Early	80	q = 0	96	90.54	101.46
TD	Nutrients_Early	80	q = 0	98	92.46	103.54
TD	Control_Late	80	q = 0	101	95.67	106.33
TD	Nutrients_Late	80	q = 0	104	97.95	110.05
TD	Control_Early	80	q = 2	34.43	32.74	36.13
TD	Nutrients_Early	80	q = 2	34.65	32.84	36.45
TD	Nutrients_Late	80	q = 2	39.47	37.02	41.92
TD	Control_Late	80	q = 2	40.04	38.06	42.02
PD	Nutrients_Early	2	q = 0	10.52	10.19	10.85
PD	Control_Early	2	q = 0	10.76	10.46	11.05
PD	Control_Late	2	q = 0	11.08	10.78	11.38
PD	Nutrients_Late	2	q = 0	11.54	11.15	11.92
PD	Nutrients_Early	2	q = 2	9.36	9.08	9.65

PD	Control_Early	2	q = 2	9.6	9.34	9.86
PD	Control_Late	2	q = 2	9.87	9.6	10.14
PD	Nutrients_Late	2	q = 2	10.27	9.93	10.61
PD	Control_Early	80	q = 0	31.49	29.03	33.96
PD	Nutrients_Early	80	q = 0	32.48	29.96	34.99
PD	Control_Late	80	q = 0	34.25	32.59	35.91
PD	Nutrients_Late	80	q = 0	35.61	33	38.22
PD	Control_Early	80	q = 2	12.75	12.36	13.15
PD	Nutrients_Early	80	q = 2	12.9	12.44	13.36
PD	Control_Late	80	q = 2	13.48	13.04	13.92
PD	Nutrients_Late	80	q = 2	14.15	13.59	14.71
FD	Nutrients_Early	2	q = 0	12.68	12.13	13.23
FD	Control_Early	2	q = 0	13.46	12.93	13.99
FD	Control_Late	2	q = 0	13.86	13.29	14.43
FD	Nutrients_Late	2	q = 0	14.22	13.68	14.75
FD	Nutrients_Early	2	q = 2	12.42	11.88	12.95
FD	Control_Early	2	q = 2	13.26	12.75	13.77
FD	Control_Late	2	q = 2	13.57	12.99	14.15
FD	Nutrients_Late	2	q = 2	13.88	13.35	14.41
FD	Control_Late	80	q = 0	15.12	14.55	15.68
FD	Nutrients_Early	80	q = 0	15.34	11.77	18.91
FD	Control_Early	80	q = 0	16.39	13.58	19.2
FD	Nutrients_Late	80	q = 0	17.66	12.92	22.39
FD	Nutrients_Early	80	q = 2	12.69	12.14	13.25
FD	Control_Early	80	q = 2	13.47	12.94	14
FD	Control_Late	80	q = 2	13.88	13.31	14.45
FD	Nutrients_Late	80	q = 2	14.24	13.71	14.78

Table S2: Effect sizes as the difference and log difference between early and late invasion for each Diversity metric, Nutrient treatment, scale, and metric Order.q . ESqd = Effect Size for Order q Diversity

Div	Nutrients	n_samples	Order.q	ESqd	Esq_Lower_CI	Esqd_Upper_CI	ESqd_log	Esqd_Lower_CI_log	Esqd_Upper_CI_log
TD	Control	2	q = 0	1.64	1.54	1.75	0.07	0.07	0.07
TD	Nutrients	2	q = 0	2.98	2.75	3.21	0.12	0.12	0.13
TD	Control	2	q = 2	1.55	1.47	1.63	0.07	0.07	0.07
TD	Nutrients	2	q = 2	2.67	2.45	2.88	0.12	0.12	0.13
TD	Control	80	q = 0	5	5.13	4.87	0.05	0.06	0.05
TD	Nutrients	80	q = 0	6	5.5	6.5	0.06	0.06	0.06
TD	Nutrients	80	q = 2	4.82	4.18	5.47	0.13	0.12	0.14
TD	Control	80	q = 2	5.6	5.32	5.89	0.15	0.15	0.15
PD	Control	2	q = 0	0.32	0.32	0.33	0.03	0.03	0.03
PD	Nutrients	2	q = 0	1.02	0.96	1.08	0.09	0.09	0.09
PD	Control	2	q = 2	0.27	0.26	0.27	0.03	0.03	0.03
PD	Nutrients	2	q = 2	0.91	0.86	0.96	0.09	0.09	0.09
PD	Control	80	q = 0	2.76	3.57	1.96	0.08	0.12	0.06
PD	Nutrients	80	q = 0	3.13	3.03	3.22	0.09	0.1	0.09
PD	Control	80	q = 2	0.73	0.68	0.77	0.06	0.05	0.06
PD	Nutrients	80	q = 2	1.25	1.16	1.35	0.09	0.09	0.1
FD	Control	2	q = 0	0.4	0.36	0.44	0.03	0.03	0.03
FD	Nutrients	2	q = 0	1.54	1.56	1.52	0.11	0.12	0.11
FD	Control	2	q = 2	0.31	0.24	0.38	0.02	0.02	0.03
FD	Nutrients	2	q = 2	1.46	1.47	1.45	0.11	0.12	0.11
FD	Control	80	q = 0	-1.27	0.97	-3.52	-0.08	0.07	-0.2
FD	Nutrients	80	q = 0	2.32	1.15	3.48	0.14	0.09	0.17
FD	Control	80	q = 2	0.41	0.37	0.45	0.03	0.03	0.03
FD	Nutrients	80	q = 2	1.55	1.57	1.53	0.12	0.12	0.11

Table S3: Effect sizes for Whittaker's beta diversity (g/a) as the difference and log difference between early and late invasion for each Diversity metric, Nutrient treatment, scale, and metric Order.q .

Div	Nutrients	Order.q	Esq	Esq_Lower_CI	ESq_Upper_CI	ESq_log	ESq_Lower_CI_log	ESq_Upper_CI_log
TD	Nutrients	q = 0	-0.28	-0.26	-0.3	-0.07	-0.06	-0.07
TD	Control	q = 0	-0.07	-0.04	-0.09	-0.02	-0.01	-0.02
TD	Nutrients	q = 2	0.01	0	0.02	0.01	0	0.01
TD	Control	q = 2	0.14	0.14	0.14	0.08	0.08	0.08
PD	Nutrients	q = 0	0	0.02	-0.02	0	0.01	-0.01
PD	Control	q = 0	0.16	0.25	0.08	0.05	0.09	0.03
PD	Nutrients	q = 2	0	0	0	0	0	0
PD	Control	q = 2	0.04	0.03	0.04	0.03	0.03	0.03
FD	Control	q = 0	-0.13	0.04	-0.29	-0.11	0.04	-0.23
FD	Nutrients	q = 0	0.03	-0.03	0.09	0.03	-0.03	0.06
FD	Nutrients	q = 2	0	0	0	0	0	0
FD	Control	q = 2	0.01	0.01	0	0.01	0.01	0

Table S4: Top 10 most abundant species, using cover values, and excluding rare species perimeter presence identification, in each treatment, quantified by summing all cover values of each species across all treatment plots

treatment	cover_sum	*Species*	Seeded	Exotic	Invasive
Control_Early_Both first	2029	*Lespedeza cuneata*	0	1	1
Control_Early_Both first	1979.5	*graminoid*	0	0	0
Control_Early_Both first	757	*Verbena urticifolia*	0	0	0
Control_Early_Both first	404	*Polygonum persicaria*	0	1	0
Control_Early_Both first	311.5	*Chenopodium album*	0	1	0
Control_Early_Both first	297	*Eupatorium altissimum*	0	0	0
Control_Early_Both first	277	*Conyza canadensis*	0	0	0
Control_Early_Both first	265	*Fallopia scandens*	0	0	0
Control_Early_Both first	264.5	*Barbarea vulgaris*	0	1	0
Control_Early_Both first	245	*Cirsium discolor*	0	0	0

Control_Late_Both first	5905	*graminoid*	0	0	0
Control_Late_Both first	1234	*Lespedeza cuneata*	0	1	1
Control_Late_Both first	1212.5	*Monarda fistulosa*	1	0	0
Control_Late_Both first	833	*Verbena urticifolia*	0	0	0
Control_Late_Both first	655	*Echinacea sp.*	NA	NA	NA
Control_Late_Both first	504	*Teucrium canadense*	0	0	0
Control_Late_Both first	489	*Conyza canadensis*	0	0	0
Control_Late_Both first	484	*Barbarea vulgaris*	0	1	0
Control_Late_Both first	481	*Acalypha virginica*	0	0	0
Control_Late_Both first	440	*Solanum carolinense*	0	0	0
Nutrients_Early_Both first	2370	*graminoid*	0	0	0
Nutrients_Early_Both first	1376	*Lespedeza cuneata*	0	1	1
Nutrients_Early_Both first	995.5	*Verbena urticifolia*	0	0	0
Nutrients_Early_Both first	647	*Polygonum persicaria*	0	1	0
Nutrients_Early_Both first	534	*Cirsium discolor*	0	0	0
Nutrients_Early_Both first	437	*Chenopodium album*	0	1	0
Nutrients_Early_Both first	434	*Teucrium canadense*	0	0	0
Nutrients_Early_Both first	275	*Conyza canadensis*	0	0	0
Nutrients_Early_Both first	246	*Solanum carolinense*	0	0	0
Nutrients_Early_Both first	209	*Barbarea vulgaris*	0	1	0
Nutrients_Late_Both first	2507	*graminoid*	0	0	0
Nutrients_Late_Both first	1505	*Monarda fistulosa*	1	0	0
Nutrients_Late_Both first	783.5	*Teucrium canadense*	0	0	0
Nutrients_Late_Both first	464.5	*Lespedeza cuneata*	0	1	1
Nutrients_Late_Both first	461.5	*Verbena urticifolia*	0	0	0
Nutrients_Late_Both first	404	*Solanum carolinense*	0	0	0
Nutrients_Late_Both first	386	*Cirsium discolor*	0	0	0
Nutrients_Late_Both first	367	*Barbarea vulgaris*	0	1	0
Nutrients_Late_Both first	260.5	*Polygonum persicaria*	0	1	0
Nutrients_Late_Both first	204.5	*Eupatorium altissimum*	0	0	0

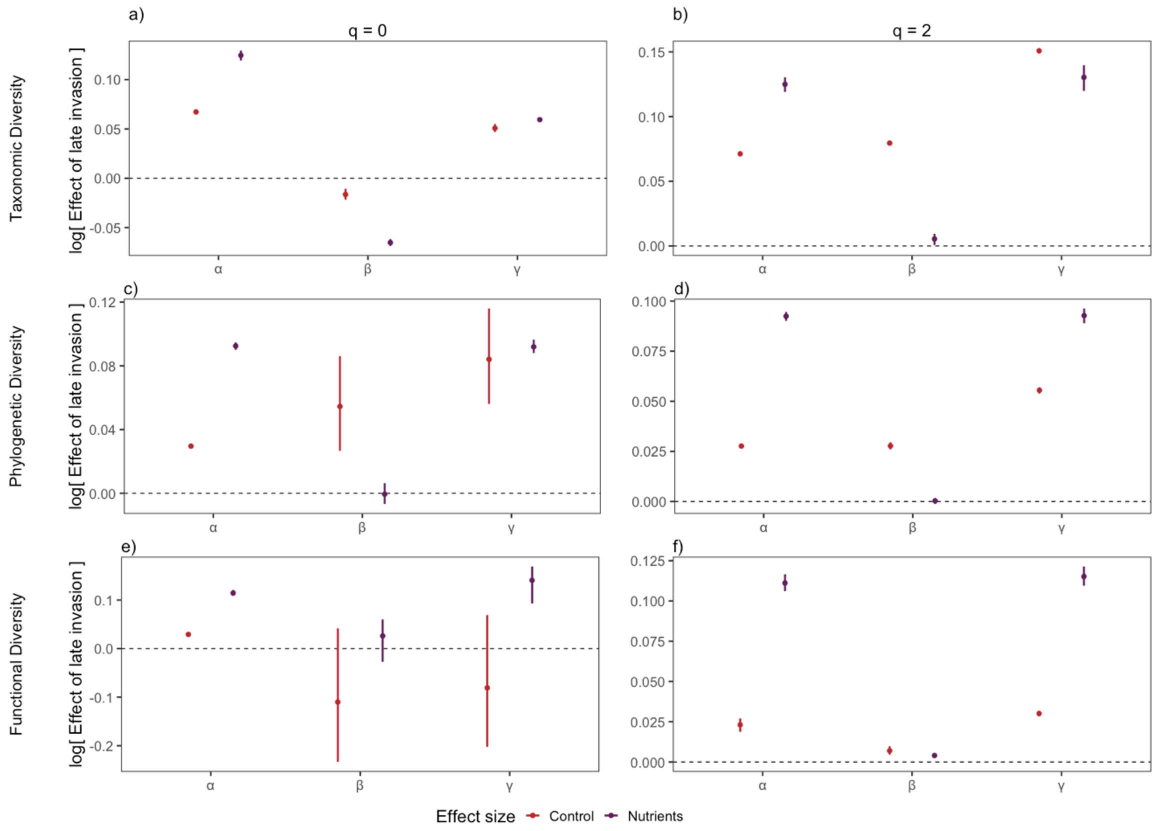

Figure S1: The log effect of Invasion timing (late - early) of *L. cuneata* on forb diversity within nutrient treatments. Points indicate mean effect; lines indicate 95% confidence intervals. Colours denote factorial nutrient treatments. Y-axes are varied for clarity.

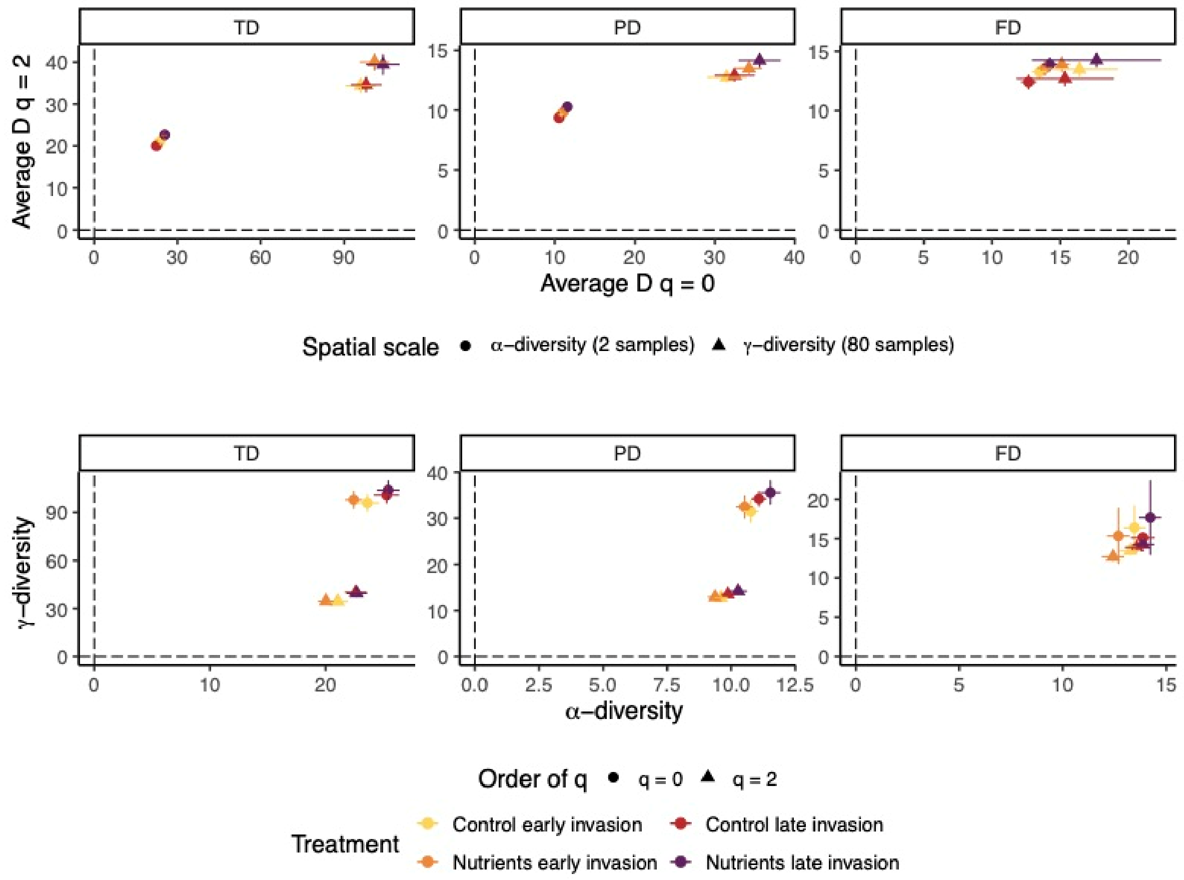

Figure S2: The joint response of diversity in top row, for forb α-diversity (x-axis) and γ-diversity (y-axis) and in bottom row, q=0 (x-axis) and q=2 (y-axis) to restoration treatments at the γ-scale (80 subplots, 20 m2). Colours denote four different factorial treatment combinations. Axes are varied for clarity.

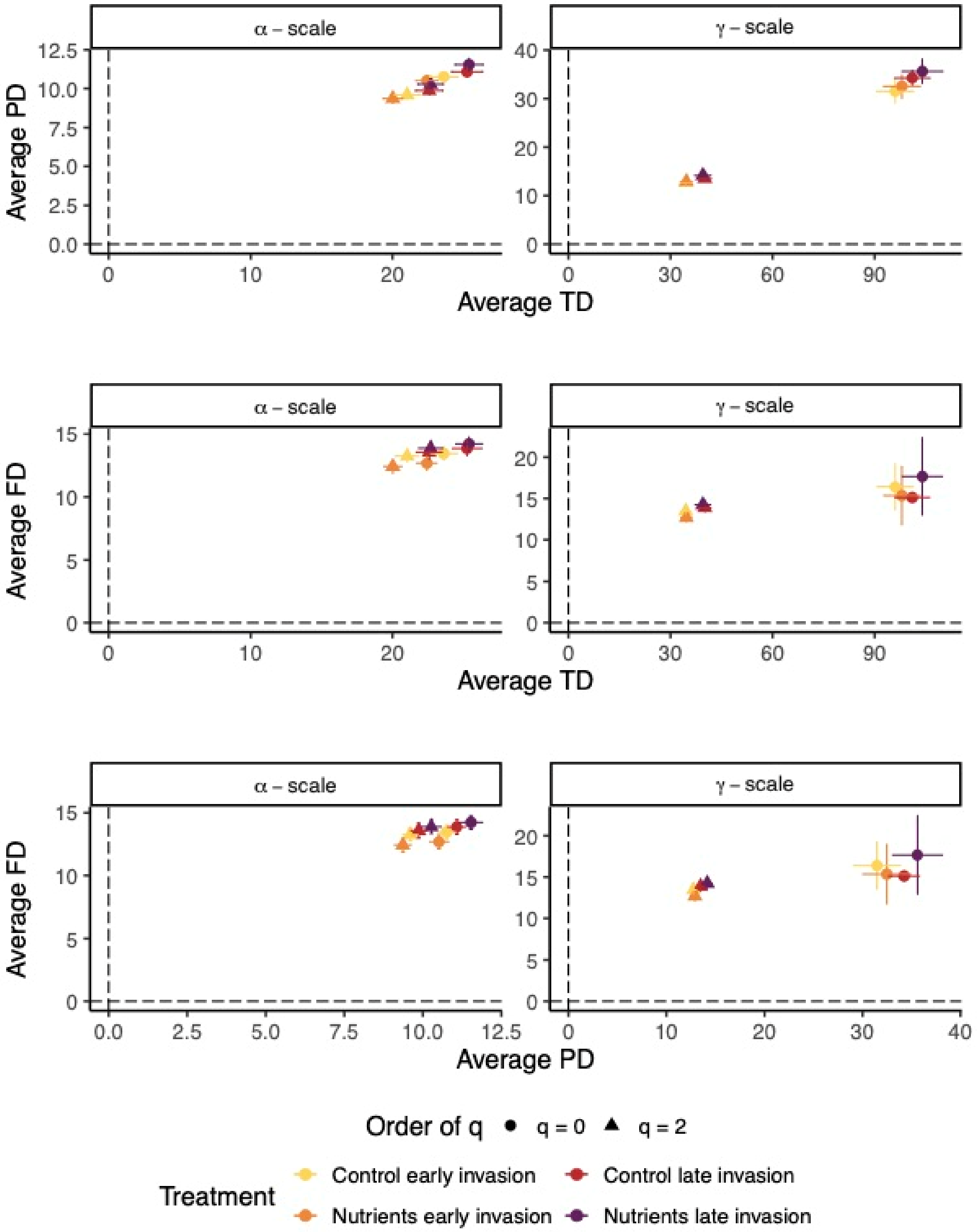

Figure S3: The joint response of forb diversity in top row, for taxonomic diversity (TD) (x-axis) and phylogenetic diversity (PD) (y-axis) for and in middle row, TD and functional diversity (FD), and bottom row, PD (x-axis) and FD (y-axis) to restoration treatments at the γ-scale (80 subplots, 20 m2). Colours denote four different factorial treatment combinations. Axes are varied for clarity.
